# How can near infrared spectroscopy be informative about the brain activation of children using a hearing implant? Preliminary case-control findings

**DOI:** 10.1590/2317-1782/e20240137en

**Published:** 2025-11-07

**Authors:** Dayanna Apolinário Diniz, Débora Marques de Miranda, Ana Lívia Libardi Bertachini, Gabriela Cintra Januário, Lurdiana Guimarães Dias, Luciana Macedo de Resende

**Affiliations:** 1 Programa de Pós-graduação em Ciências Fonoaudiológicas, Faculdade de Medicina, Universidade Federal de Minas Gerais – UFMG - Belo Horizonte (MG), Brasil.; 2 Programa de Pós-graduação em Ciências da Saúde da Criança e do Adolescente, Departamento de Pediatria, Faculdade de Medicina, Universidade Federal de Minas Gerais – UFMG - Belo Horizonte (MG), Brasil.

**Keywords:** Hearing, Cochlear Implant, Hearing Loss, Spectroscopy, Near-Infrared, Plasticity

## Abstract

The auditory rehabilitation mechanism with cochlear implants can activate specific cortical regions of the brain similar to those of hearing people. Near-infrared spectroscopy (fNIRS) is a neuroimaging tool that makes it possible to evaluate the cortical development of implanted children. Objective: To investigate the molecular imaging response, reflected by difference in cortical oxygenation, in a deaf child with a cochlear implant compared to hearing children, observe and report auditory performance verifying correspondence with fNIRS findings. Methods: Comparative case study using the fNIRS technique to evaluate the child's cortical activation on the day of device activation with three hearing children of the same hearing age. The recorded data was processed with Brainstorm software and statistical analysis was performed with the Power F test in relation to the baseline and the Permutation test for comparison. Longitudinal analysis of the implanted child's audiological data was carried out, as well as auditory perception and language questionnaires. Results: No statistical differences were found between cortical activation of the implanted child and hearing children of the same hearing age. An improvement in the child's auditory perception and language performance can be observed after using the implant. Conclusion: The evolution of auditory responses was observed, showing cortical maturation after activation of the cochlear implant seen in the molecular image, concomitantly with the use of hearing aids and speech therapy. It was observed that fNIRS is a neuroimaging technique capable of recording cortical changes in children with and without implants, being a promising tool.

## INTRODUCTION

A cochlear implant (CI) is a device indicated for the rehabilitation of severe to profound hearing loss, enabling sound perception and reintegration into the social world of hearing^(^1^)^. It is very important that this procedure be performed at an appropriate time, within a sensitive period, to promote hearing and language development^(^2^)^.

Many children with CI will eventually achieve language comprehension and vocabulary equivalent to normal-hearing children of the same age, but a significant proportion (68%) do not show good results after using the implant^(^3^)^. Studies suggest that central plasticity may be an important factor in the different levels of benefit that implanted individuals experience^(^4,5^)^.

The human cortex demonstrates the capacity for neuroplasticity during its development, in accordance with changes in sensory input. Thus, sensory deprivation causes anatomical and functional changes in the brains of individuals with hearing deprivation^(^6^)^.

The process of neural connection development, known as functional synaptogenesis, peaks in the auditory cortex around 2 to 4 years of age, which is the critical period of cortical organization. The immature brain during the period of plasticity is especially ideal for benefiting from cochlear implants^(^7,8^)^.

Cochlear implant devices contain electrodes placed inside the cochlea that directly stimulate the spiral ganglia of the auditory nerve. Auditory perception occurs within and beyond the auditory cortex. Understanding how this mechanism of electrical stimulation of the cochlea by the CI activates specific auditory cortical regions of the brain similar to those of normal-hearing individuals is a major challenge^(^3,9^)^.

Because of this, objective neuroimaging tests can be a useful tool for understanding how much auditory and speech information is received by the brain of a child with an implant and in which regions this stimulus is being processed, with a view to understanding the behavior of implanted individuals.

In this context, Near-InfraRedSpectroscopy (fNIRS) is an optical imaging technique that uses near-infrared light to detect changes in blood flow in the cerebral cortex. This technique uses sources and detectors that capture the molecular response of the tissue, related to the concentration of oxygenated hemoglobin (HbO), deoxygenated hemoglobin (dHb), and total hemoglobin (tHb)^(^6,9^)^.

When a region of the brain is activated, there is an increase in metabolic demand, leading to increased blood flow to oxygenate that region. This method of measuring hemoglobin change acts as a substitute/correlate for neural activity^(^10^)^. This approach has proven to be safe and appropriate for use in assessing hearing and language development in implanted children^(^11^)^.

This study aimed to investigate the molecular imaging response, reflected by the difference in cortical oxygenation, in a deaf child with a cochlear implant compared to hearing children of the same hearing age, and to observe and report hearing performance by verifying the correspondence between sensory speech perception performance using NIRS.

## PRESENTATION OF THE CLINICAL CASE

This is a comparative case study between a child with profound sensorineural hearing loss and hearing children of the same hearing age.

The study was approved by the Research Ethics Committee of the educational institution under opinion no. 3.340.222 and followed the recommendations of resolution 466/12 of the CNS.

### Case characterization

A nine-months female child arrived at the pediatric hearing health service. The parents sought care after noticing that the child was not responding to sound stimuli. During the anamnesis, the mother reported that there were no complications during pregnancy and delivery, the child was born at term by cesarean section, with an Apgar score of eight at one minute and nine at five minutes, weight and height appropriate for gestational age, and was discharged two days after birth. According to the information collected, there were no risk indicators for hearing loss. The result of the neonatal hearing screening was absence of V wave at 35 dBnHL in the automatic brainstem auditory evoked potential test.

### Diagnostic procedures:

For the audiological diagnosis, the following tests were performed: Transient Evoked Otoacoustic Emissions (TEOAE) with no responses, Brainstem Auditory Evoked Potential (BAEP) click with no wave formation bilaterally at 90 dBnHL, Immitance measures with bilateral type A tympanometric curve and absence of ipsilateral and contralateral acoustic reflexes; Auditory Steady State Responses (ASSR) with the results described in Table 1. In visual reinforcement audiometry, responses were absent for stimuli of 80dBSPL (maximum output of the audiometer).

**Table 1:** Electrophysiological thresholds obtained in steady-state auditory evoked potentials in dBnHL

	**500Hz**	**1Hz**	**2Hz**	**4Hz**
**RE**	100dBnHL	100dBnHL	100dBnHL	100dBnHL
**LE**	90dBnHL	90dBnHL	90dBnHL	90dBnHL

Caption: RE = right ear; LE = left ear

Based on the results, the child was diagnosed with profound bilateral sensorineural hearing loss. The patient was referred for bilateral hearing aids fitting and specialized speech therapy.

As there were no risk factors for hearing loss, it is believed to be an autosomal recessive genetic disorder (under investigation).

### Audiological intervention approach

The patient was fitted with hearing aids at 10 months of age. In the first assessment, the patient's average in situ gain (REIG) with the device was 42 dB. In the second adjustment of the fitting, the gain was increased to 51 dB. During this period, the child began specialized speech therapy once a week, but no significant improvements were observed.

In the Behavior Observation Audiometry, performed after one month of using the hearing aids, there was no response to uncalibrated instrumental sounds of medium and high intensity. Due to the audiological diagnosis, lack of benefit from using the device, age, and family motivation, a cochlear implant was prescribed.

At one year and four months of age, the child underwent cochlear implant surgery in the right ear. She received an Advanced Bionics (AB) HiRes 90k Advantage device. The surgery was uneventful, with full insertion of the electrodes into the cochlea. Impedance telemetry showed responses within normal limits in all electrodes. Intraoperative neurotelemetry was performed on four electrodes (E3, E7, E11, and E15), with responses also present in all tested electrodes.

During surgery and at the time of activation, electrode impedances were tested, with responses within normal limits in all channels.

### Cochlear implant activation

One month after surgery, the implant was activated, and the Naída CI Q70 sound processor, manufactured by AB, was fitted, maintaining the use of the hearing aid in the contralateral ear. Five progressive maps were created based on observation of the child's behavioral responses, ensuring the use of audible and comfortable safety levels.

In the auditory behavior observation performed on the day of activation, with bimodal adaptation, hearing aid, and CI, lateral localization to the right and left was observed for medium-intensity instrumental sounds (rattle and *reco-reco*); cochleopalpebral reflex (CPR) for *agogô* sounds and reaction to voice at usual intensity. In the visual reinforcement audiometry (VRA) assessment, the minimum response levels found were: 500Hz – 45dB, 1000Hz – 60dB, 2000Hz – 65dB, 4000Hz – 70dB.

In addition, the child responded to her name being called, paid attention to voice sounds and knocking on the door, and detected the sounds /a/, /i/, and /u/, but did not detect the sounds /s/, /ʃ/, and /m/ during the Ling test.

On this occasion, based on the speech-language evaluation, the child was classified as hearing category 1 and language category 1.

### Characterization of controls

For comparison purposes, three healthy children (according to medical records and reports from their guardians) participated in this study. They were matched according to the hearing age of the implanted patient and had no risk indicators for hearing loss, with the presence of a V wave at 35 dBnHL on neonatal hearing screening. All children underwent hearing assessment, including acoustic immittance measurements, automatic brainstem auditory evoked potential at 35 dBnHL (AABR) with CE-chirp stimulus, and otoacoustic emissions evoked by transient stimuli (TEOAE), with results compatible with normal standards for the age group.

### fNIRS collection protocol

After CI activation, on the same date, fNIRS was recorded to assess brain activation in response to auditory stimulation using the device, as described below.

**Figure 1:**
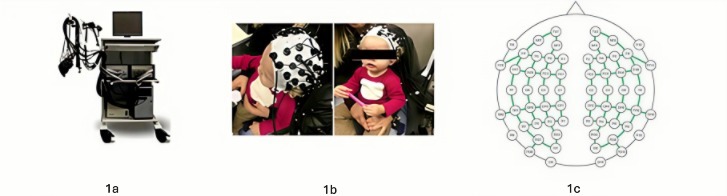
Data recording with fNIRS

**Figure 2:**
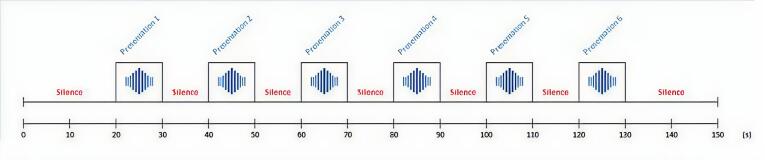
Visual representation of the presentation of auditory stimuli over time

The near-infrared spectroscopy (fNIRS) technique was used to investigate cortical hemodynamic activity. The equipment used for the study was the NIRScout Tandem 1616 (NIRx Medical Technologies, Glen Head). The protocol developed used light source wavelengths of 760 and 850 nm, with 30 sources and 28 detectors, forming 84 channels covering the frontal, temporal, parietal, and occipital lobes bilaterally, using the international 10-20 system as a reference for optode placement. The distances between the sources and detectors were approximately 3.5 cm. Figures 1 and 2 illustrate the collection protocol developed for this study.

Caption: 1a: Device used for fNIRS data acquisition; 1b: Picture recording personal cortical activation; 1c: Geometry used for the deployment of spectroscopy test channels

Caption: In red – cortical areas activated during stimulation

In the geometry that was used, the 84 channels are represented by green lines.

To collect the children's cortical responses to the sound stimulus, the protocol consisted of blocks of maternal voice stimulation in the form of spontaneous conversation with prosody directed at the child. Sound stimulation occurred six times with an average duration of 10 seconds each, interspersed with 10-second intervals of silence (Figure 2). During the task, the children were held in their mothers' laps, and the intensity of the vocal emission was around 60 dBSPL, measured with a handheld decibel meter.

### fNIRS data preprocessing protocol

The recorded data were processed in *Brainstorm*, an open-source application for analyzing brain recordings such as MEG, EEG, and fNIRS^(^12^)^. The recording was processed based on the general protocol used in previous fNIRS experiments^(^13-15^)^.

To extract the hemodynamic response function (HRF) for each channel and reject those that did not provide sufficient data due to noise or motion artifacts^(^13^)^, it was necessary to apply a 10-step procedure to the recordings, as described below: (1) Manually detect and mark motion artifacts characterized by signal variation with peaks that spread to the channels and start semi-automatic motion correction based on spline interpolation^(^16^)^; (2) Automatically detect bad channels due to negative or paired values or due to many flat segments using a maximum ratio of saturation points and floor equal to 1.00; (3) Remove glitches (variation threshold 2 times the standard deviation); (4) Calculate variations in oxyhemoglobin (HbO), deoxyhemoglobin (dHb), and total hemoglobin (tHb) concentrations using the modified Beer-Lambert Law and configure the process options with a reference age of 1 year, partial volume factor (PVF) equal to 6, DPF method^(^17^)^, and based on the average; (5) Automatically remove the linear trend; (6) Use a 0.02Hz and 0.8Hz bandpass filter to reduce slow systemic hemodynamic and physiological fluctuations and high-frequency instrument noise and rapid oscillations; (7) Apply a notch filter to eliminate power line signal contamination at 30Hz; (8) Automatically detect and exclude channels using MATLAB codes when intensity changes have a low signal-to-noise ratio (between the standard deviation and the mean greater than 5 in a 5-second time window to avoid inadequate recording due to thick hair^(^13^)^; (9) Visually inspect the data and remove channels with high amplitude and signal variations during block recording; and; (10) Visually inspect all data and remove those that still show movement artifacts from the analysis.

The time window used ranged from -10,000 ms to +15,000 ms from the start of the stimuli marked by the triggers. To establish a standard, all samples were adjusted to a sampling range of 4.0 Hz. For baseline normalization, the study used Z-score transformation, selecting the baseline definition from -5 to 0s. Finally, the mean hemodynamic response function was calculated for each child.

The modified Beer-Lambert law was used to analyze hemoglobin and deoxyhemoglobin concentrations in response to cortical activation. The law consists of an equation that describes the absorption of light at a given wavelength in a milieu. The law was modified to take into account the dispersion and nonlinear trajectory of light in tissues. These two factors cannot be measured using fNIRS, so only changes in HbO and dHb concentrations can be obtained^(^14^)^.

For statistical analysis, the Power F test was used against the baseline, where Y = sum_trials(X^2) and F = Y/mean time (Y (baseline). The aim was to find significant changes, relative to the baseline, in HbO and dHb concentrations in response to auditory stimuli, regardless of whether the values were negative or positive.

The response of the implanted child was analyzed with three hearing children of the same hearing age. To compare the groups, the Permutation test was used, using the absolute mean test (i.e., T = (|mean(A)|-|mean(B)|) / sqrt(|var(A)|/nA + |var(B)|/nB)).

### Comparative fNIRS results of the implanted child and hearing children

The responses were calculated considering the HbO and dHb measurements resulting from cortical activity in response to changes in blood oxygenation when processing the stimuli offered. The following images show the response to auditory stimuli presented through the mother's voice.

**Figure 3:**
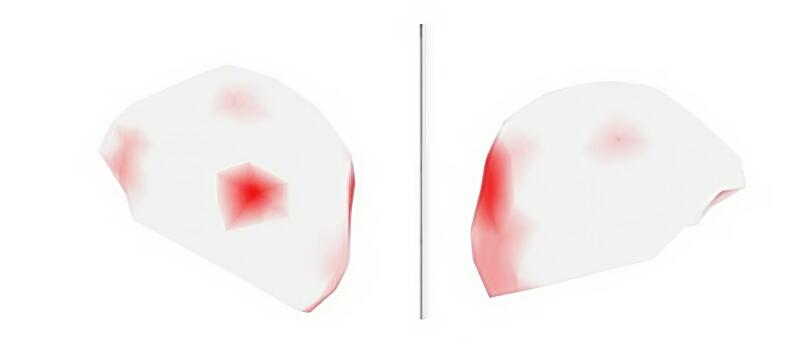
Power test of the implanted child's activation in response to the sound stimulus of the mother's voice

Figure 3 shows the power test of the child using the cochlear implant (case) with the recording of activation in response to auditory stimulation of the mother's voice. The red dots represent the activated points.

Caption: Comparison between implanted children and hearing children – no significant difference

Greater activation can be observed in the temporal and parieto-occipital regions. After analyzing the response of the implanted child, the power test was used to compare brain activity between the groups.

**Figure 4:**
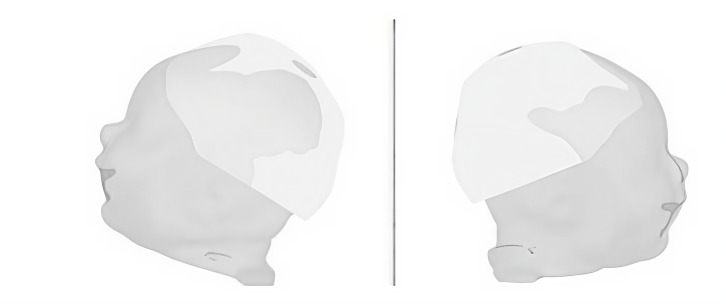
Power test of the group of three control children of the same hearing age

Figure 4 shows the power test comparing the response of the implanted child with the response of three children of the same hearing age to the mother's voice stimulus.

The blank image, provided by the software, shows that there was no difference from the baseline (either positive or negative) in this group. This suggests that in the group of hearing children, there was no difference between the stimulation period and the baseline of this same group.

## DISCUSSION

In the present study, we observed hearing performance after cochlear implant adaptation in a child with profound sensorineural hearing loss and also the dynamics of the activated brain areas using neuroimaging. The cortical hemodynamic response of the child with hearing loss was compared with the cortical response of three hearing children of the same hearing age.

The recordings made by near-infrared spectroscopy showed greater activation in the temporal region in both hemispheres and also in the occipital region. Generally, the temporal region is activated to process prosody and speech, and the occipital region is related to the processing of visual stimuli^(^11^)^. Studies show that children who receive early implantation activate the same cortical areas as individuals with normal hearing in response to sounds, whereas children who receive late implantation activate other cortical areas unrelated to auditory areas when they hear a sound^(^18^)^_._ This can be considered a diffuse pattern of cortical response due to the sensory limitation imposed by hearing loss.

Neuronal plasticity is shaped by the demands of the environment^(^2,18^)^. There are different sensitive periods for different behavioral functions, due to cortical structures and functions^(^18^)^. The child in the study received a cochlear implant at 1 year and 4 months, within the period considered sensitive by the literature^(^19^)^ and without significant structural changes in the cortex, as observed by comparison with fNIRS.

During this period, neuronal plasticity and maturation of the auditory pathways occur, enabling the acquisition of hearing, speech, and language skills. In the clinical case of the study, we observed that the child began the process of restoring hearing function at a time conducive to cortical development, i.e., before reaching 12 months of age.

Brain development occurs through the stages of neurogenesis, neuronal migration, neuronal development, elimination, and formation of central pathways, generating a functional brain connection. The lack of sound stimulation of the auditory cortex can lead to functional decoupling of the primary cortex and secondary auditory areas^(^2,20^)^. The lack of sensory input has consequences from the beginning of life. Auditory stimulation through cochlear implantation during the sensitive period induces plasticity and maturation, reducing the negative consequences for neurodevelopment^(^11,21^)^.

Auditory information is processed in the brain through the analysis and categorization of the acoustic characteristics of sound. These functions are subjective and depend on the learning process. Sensory stimulation favors the reorganization of the receptive fields of the cerebral cortex, acquiring meanings. The new brain has greater capacity for reorganization and neuronal plasticity compared to already developed brains^(^19,22^)^.

When comparing the activation response of the implanted child with that of hearing children, the response pattern was similar, allowing us to infer that even with the activation of the cochlear implant, when the target hearing level had not yet been achieved, the child showed cortical hemodynamic responses similar to those of his peers with normal hearing and the same hearing age. Although the case presented here involves prelingual hearing loss, where the phenomenon of cross-modality could have caused structural changes in the child's cortex, the similarity of responses compared to their hearing peers demonstrates how important intervention is during the sensitive period for the best development of the child's hearing and language functions.

A population study of children with congenital deafness showed that the benefit of cochlear implantation was evident when surgery occurred between the first 3.5 years of life, when the central auditory pathways show maximum plasticity. To confirm the data, cortical auditory evoked potential assessments were performed, which allowed observation of the latency and morphology of the P1 component (considered a biomarker of cortical maturation). There was a decrease in P1 latency after cochlear implantation, and the results were similar to those of hearing children. In addition, the same researchers observed that children who received implants before the age of 3-4 years performed better in speech perception and language skills when compared to children implanted after the age of 6-7 years^(^23^)^.

It is also important to note that the child under study, in addition to using a cochlear implant, uses a contralateral individual sound amplification device, promoting a bimodal adaptation system, that is, the use of electrical stimulation via a cochlear implant associated with acoustic stimulation in the other ear. As reported in the literature, this adaptation model provides benefits related to speech perception in noise, sound localization, and other communication situations. Due to the binaural summation effect, the brain perceives sound as more intense, requiring less concentration and greater ease of listening by the individual^(^2^)^.

Although subjective tests are widely used in clinical practice and have proven reliable for measuring the hearing and language skills of deaf children who use individual sound amplification devices or cochlear implants, understanding what happens at the cortical level in these individuals can broaden knowledge about auditory development with the use of devices and speech therapy.

Studies show that children who receive early implantation activate the same cortical areas as individuals with normal hearing in response to sounds, while children who receive late implantation activate other areas of cortical activation unrelated to auditory areas when they hear a sound^(^18^)^_._

The understanding of how brain activity occurs and how it can influence CI outcomes could not only help predict the success of this procedure, but also monitor postoperative responses in these patients, helping to guide the search for better results^(^5,6^)^.

The use of fNIRS provides access to information about cerebral oxygenation, especially in areas of gray matter with an average depth of 3-4 cm^(^11,24^)^. fNIRS allows demonstrating the functional aspect of activation of a specific brain region in response to a specific stimulus. As it is a technology based exclusively on light, there is no risk to the patient, and the limitations consist only of electrode fixation and establishing a good signal in the capture^(^6^)^.

Neuroimaging, visualized from the analysis of the captured cortical responses, assists in monitoring auditory performance with CI, as auditory perception occurs beyond the auditory cortex, and the molecular image, generated by changes in cerebral oxygenation (cortical hemodynamics), provides additional clinical information to assess whether electrical stimulation of the CI in the cochlea is reaching and stimulating specific auditory cortical regions of the brain similar to what occurs in individuals with normal hearing^(^24,25^)^.

In a study using fNIRS in CI users, speech-evoked cortical responses were observed in four groups: adults with normal hearing, children with normal hearing, deaf children who had more than four months of hearing through a cochlear implant, and deaf children one month after implantation. The speech stimuli consisted of recordings of children's stories in English. The fNIRS data were compared with data found on functional magnetic resonance imaging and found similar responses of superior temporal gyrus activation. These results demonstrated that fNIRS was a viable neuroimaging technique in CI users and that reliable hemodynamic cortical responses to speech could be recorded in these patients^(^26,27^)^.

In a study geared to assess the sensitivity of fNIRS to detect differences in cortical activation in response to speech quality in normal individuals, a 140-channel fNIRS system (NIRScout, NIRx Medical Technologies LLC, Glen Head, NY) was used. The protocol used consisted of four different stimuli: normal speech, channeled speech (simulating the channel distribution of the cochlear implant), scrambled speech, and ambient noise. As results, the researchers observed that normal speech elicited the strongest responses, distorted speech produced less specific activation of the region, and environmental sounds evoked the lowest response, making it possible to state that fNIRS can detect differences in intelligibility response in hearing individuals^(^10^)^. Based on this study, an experimental study was conducted involving participants with CI. The NIRScout 1624 instrument (NIRx Medical Technologies, LLC, Glen Head, NY) with 140 channels was used to record the cortical auditory response of 32 post-lingually deaf adults listening through a CI and 35 adults with normal hearing. The same stimuli were adopted as protocol: normal speech, channeled speech, scrambled speech, and ambient noise. In this experiment, it was possible to observe that the cortical activation pattern in implanted adults with good speech perception was similar to that of the controls. The results of this study demonstrated that the activation patterns in the auditory cortex of CI recipients are correlated with the quality of speech perception. The assessment was repeated with the implant turned off, and reduced cortical activations were observed in all CI recipients^(^10^)^.

The fNIRS technique and recording can also be performed in conjunction with electroencephalography, and a study with implanted children identified that the cross-modal response in these children is not as specialized and uniform as expected^(^28^)^.

In hearing children, even in infants, bilateral cortical activation is observed in response to sound stimulation, and responses to the mother's voice are still diffuse and indistinguishable from other stimuli^(^29^)^.

All these studies show how widely cortical responses can be used to deepen our understanding of auditory development.

New research related to the evaluation of NIRS concomitant with the use of cochlear implants should be developed.

Considering the various techniques for collecting, processing, and analyzing results found in the research conducted, it is necessary to develop protocols to validate NIRS recordings and interpretations with CI, facilitating the understanding of counseling and rehabilitation for patients before and after implantation^(^10^)^. In the future, fNIRS may become a pre- and post-implantation test to provide important clinical information related to patient prognosis and intervention, supporting individual therapies and enhancing their results^(^24^)^.

## FINAL CONSIDERATIONS

This comparative case report described auditory responses after activation of a cochlear implant in concomitant use with a hearing aid. Cortical responses were observed in order to understand the changes that occurred in the brain of a child with hearing loss who had an implant, compared to the brain of hearing children.

The results suggest once again that timely cochlear implantation, when the cortex is not yet fully specialized to process certain tasks, is important for maximizing neuronal plasticity, favoring the development of hearing and language skills.

In addition, fNIRS has again proven to be an interesting tool for use in cochlear implant users.

The application of these findings in the pediatric population using cochlear implants is very promising for detecting and monitoring the prognosis and rehabilitation of these individuals. The study is being expanded to include a larger number of children and various matching conditions for comparative purposes. Its methodology can be replicated in other studies with pediatric populations to observe auditory development resulting from auditory rehabilitation.
