# Supplementary material for: How can near infrared spectroscopy be informative about the brain activation of children using a hearing implant? Preliminary case-control findings
Source: Codas. 2025 Nov 7;37(5):e20240137. doi: 10.1590/2317-1782/e20240137en (PMC12617279; doi:10.1590/2317-1782/e20240137en)
Supplement: Translation

# Translation: Como a espectroscopia de luz no infravermelho próximo pode ser informativa sobre ativação cortical em crianças usuárias de implante coclear? Achados preliminares em estudo de caso-controle

**DOI:** 10.1590/2317-1782/e20240137pt

**Authors:** Dayanna Apolinário Diniz, Débora Marques de Miranda, Ana Lívia Libardi Bertachini, Gabriela Cintra Januário, Lurdiana Guimarães Dias, Luciana Macedo de Resende

**Affiliations:** 1 Programa de Pós-graduação em Ciências Fonoaudiológicas, Faculdade de Medicina, Universidade Federal de Minas Gerais – UFMG - Belo Horizonte (MG), Brasil.; 2 Programa de Pós-graduação em Ciências da Saúde da Criança e do Adolescente, Departamento de Pediatria, Faculdade de Medicina, Universidade Federal de Minas Gerais – UFMG - Belo Horizonte (MG), Brasil.

**Keywords:** Audição, Implante Coclear, Perda Auditiva, Espectroscopia de Luz Próxima ao Infravermelho, Plasticidade

## Abstract

A reabilitação auditiva com o implante coclear pode ativar regiões corticais específicas do cérebro semelhantes àquelas de pessoas ouvintes. A espectroscopia de luz no infravermelho próximo (fNIRS), uma ferramenta de neuroimagem, possibilita avaliar o desenvolvimento cortical de crianças implantadas. Objetivo: investigar a resposta de imagem molecular, refletida pela diferença de oxigenação cortical, em uma criança surda com implante coclear em comparação com crianças ouvintes de mesma idade auditiva, observar e relatar o desempenho auditivo verificando a correspondência entre o desempenho da percepção auditiva sensorial da fala usando a fNIRS. Métodos: Estudo de caso comparativo com o uso da técnica fNIRS para avaliar a ativação cortical da criança no dia da ativação do dispositivo com três crianças ouvintes de mesma idade auditiva. Os dados registrados foram processados em Brainstorm e a análise estatística foi realizada com Power F em relação à linha de base e o teste Permutation para comparação. Realizada a análise longitudinal dos dados audiológicos da criança implantada, questionários de percepção auditiva e de linguagem. Resultados: Não foram encontradas diferenças estatísticas entre ativação cortical da criança implantada com crianças ouvintes de mesma idade auditiva. Pode-se observar melhora do desempenho da percepção auditiva e da linguagem da criança após uso do implante. Conclusão: Observou-se a evolução das respostas auditivas evidenciando a maturação cortical após a ativação do implante coclear visto na imagem molecular, concomitante ao uso do AASI e à fonoterapia. A fNIRS é capaz de registrar mudanças corticais em crianças com e sem implante, sendo uma ferramenta promissora.

## INTRODUÇÃO

O implante coclear (IC) é um dispositivo indicado na reabilitação das perdas auditivas de grau severo a profundo, possibilitando a percepção sonora e a reintegração para o mundo social da audição^(1)^. É muito importante que esse procedimento seja realizado em um tempo oportuno, dentro de um período sensível, para favorecer o desenvolvimento da audição e linguagem^(2)^.

Muitas crianças com IC, em algum momento, alcançarão a compreensão de linguagem e vocabulário equivalente a crianças com audição normal da mesma idade, porém, nota-se que uma proporção significativa (68%) não apresenta bons resultados após o uso do implante^(3)^. Estudos sugerem que a plasticidade central pode ser um fator importante nos diferentes níveis de benefício que os indivíduos implantados apresentam^(4,5)^.

O córtex humano demonstra a capacidade de neuroplasticidade durante seu desenvolvimento, de acordo com alterações na entrada sensorial. Assim, a privação sensorial causa alterações anatômicas e funcionais nos cérebros em privação auditiva^(6)^.

O processo de desenvolvimento de conexões neurais, conhecido como sinaptogênese funcional, atinge o pico no córtex auditivo por volta dos 2 a 4 anos de idade, sendo esse o período crítico de organização cortical. O cérebro imaturo durante o período de plasticidade é especialmente ideal para se beneficiar do implante coclear^(7,8)^.

Os dispositivos de implante coclear contém eletrodos colocados dentro da cóclea que estimulam diretamente gânglios espirais do nervo auditivo. A percepção auditiva ocorre dentro e além do córtex auditivo. Entender como esse mecanismo de estimulação elétrica da cóclea pelo IC ativa regiões corticais auditivas específicas do cérebro semelhantes àquelas de pessoas normo-ouvintes é um grande desafio^(3,9)^.

Devido a isso, testes objetivos de neuroimagem podem ser uma ferramenta útil para entender o quanto de informação auditiva e de fala é recebido pelo cérebro da criança com implante e em que regiões esse estímulo está sendo processado, visando compreender o comportamento dos indivíduos implantados.

Nesse contexto, a espectroscopia de luz no infravermelho próximo (*Near Infrared Spectroscopy -* fNIRS) é uma técnica de imagem óptica, que utiliza a luz infravermelha próxima para detectar alterações do fluxo sanguíneo no córtex cerebral. Essa técnica utiliza fontes e detectores que captam a resposta molecular do tecido, relacionadas a concentração de hemoglobina oxigenada (HbO), desoxigenada (HbR) e hemoglobina total (HbT)^(6,9)^.

Quando uma região do cérebro é ativada, ocorre um aumento da demanda metabólica levando ao aumento do fluxo sanguíneo para oxigenar essa região. Esse método de medir a alteração da hemoglobina atua como substituto/correlato da atividade neural^(10)^. Essa abordagem provou ser segura e apropriada para uso na avaliação do desenvolvimento auditivo e de linguagem em crianças implantadas^(11)^.

Este estudo teve como objetivo investigar a resposta de imagem molecular, refletida pela diferença de oxigenação cortical, em uma criança surda com implante coclear em comparação com crianças ouvintes de mesma idade auditiva, observar e relatar o desempenho auditivo verificando a correspondência entre o desempenho da percepção auditiva sensorial da fala usando a NIRS.

## APRESENTAÇÃO DO CASO CLÍNICO

Esse é um estudo de caso comparativo, entre uma criança com perda auditiva neurossensorial de grau profundo, em relação a crianças ouvintes de mesma idade auditiva.

O estudo foi aprovado pelo Comitê de Ética em Pesquisa da instituição de ensino sob parecer no. 3.340.222 e seguiu as recomendações da resolução 466/12 do CNS.

### Caracterização do caso

Criança do sexo feminino, chegou ao serviço de saúde auditiva infantil aos nove meses de idade. Os pais procuraram o atendimento após perceberem que a criança não respondia aos estímulos sonoros. Durante a anamnese, a mãe relatou que não houveram intercorrências na gestação e parto, a criança nasceu a termo de parto cesárea, apgar oito no primeiro minuto e nove no quinto minuto, peso e estatura adequados à idade gestacional e recebeu alta após dois dias do nascimento. De acordo com as informações coletadas, não apresenta indicador de risco para perda auditiva. O resultado da triagem auditiva neonatal foi ausência de onda V a 35 dBnNA na testagem do potencial evocado auditivo de tronco encefálico automático.

### Procedimentos diagnósticos

Para o diagnóstico audiológico, foram realizados os seguintes exames: Emissões Otoacústicas Evocadas por estímulos transientes (EOAT) com ausência de respostas, Potencial Evocado Auditivo de Tronco Encefálico (PEATE) clique com ausência de formação de ondas bilateralmente em 90 dBnNA, Imitanciometria com curva timpanométrica do tipo A bilateralmente e ausência de reflexos acústicos ipsi e contralaterais; Potenciais Evocados Auditivos de Estado Estável (PEAEE) com os resultados, descritos na Tabela 1. Na audiometria de reforço visual, as respostas foram ausentes para estímulos de 80dBNPS (saída máxima do audiômetro).

**Tabela 1:** Limiares eletrofisiológicos obtidos no Potencial evocado auditivo de estado estável em dBnNA

	**500Hz**	**1Hz**	**2Hz**	**4Hz**
**OD**	100dBnNA	100dBnNA	100dBnNA	100dBnNA
**OE**	90dBnNA	90dBnNA	90dBnNA	90dBnNA

Legenda: OD = orelha direita; OE = orelha esquerda

De acordo com os resultados encontrados, a criança foi diagnosticada com perda auditiva neurossensorial de grau profundo bilateral. Como conduta foi realizado o encaminhamento para adaptação de AASI bilateral e terapia fonoaudiológica especializada.

Por não haver indicador de risco para perda auditiva acredita-se ser uma perda de origem genética autossômica recessiva (em investigação).

### Abordagem de intervenção audiológica

A paciente foi adaptada com o aparelho de amplificação sonora individual aos 10 meses de idade. Na primeira avaliação, o ganho médio in situ (REIG) da paciente com o dispositivo foi de 42dB. Na segunda regulagem da adaptação o ganho foi aumentado para 51dB. Nesse período, a criança iniciou fonoterapia especializada uma vez por semana, porém não foram observadas melhorias significativas.

Na Observação do Comportamento Auditivo, realizada após um mês de uso do AASI, observou-se ausência de respostas para sons instrumentais não calibrados de média e forte intensidade. Devido ao diagnóstico audiológico, falta de benefício com o uso do dispositivo, idade e pela motivação da família, foi realizada a indicação do implante coclear.

Com um ano e quatro meses de idade, a criança foi submetida à cirurgia do implante coclear na orelha direita. Assim, recebeu o dispositivo da marca *Advanced Bionics* (AB), modelo *HiRes 90k Advantage*. A cirurgia ocorreu sem intercorrências, com inserção total dos eletrodos na cóclea. A telemetria de impedâncias apresentou respostas dentro dos limites de normalidade em todos os eletrodos. A neurotelemetria intraoperatória foi realizada em quatro eletrodos (E3, E7, E11 e E15), com respostas também presentes em todos os eletrodos testados.

Durante a cirurgia e no momento da ativação, foram testadas as impedâncias dos eletrodos, com respostas dentro dos limites de normalidade em todos os canais.

### Ativação do implante coclear

Após um mês da cirurgia, realizou-se a ativação do implante, e foi adaptado o processador de som Naída CI Q70, marca AB, mantendo o uso do AASI na orelha contralateral. Foram criados cinco mapas progressivos a partir da observação das respostas comportamentais da criança, certificando-se de utilizar níveis de segurança audíveis e confortáveis.

Na observação do comportamento auditivo realizada no dia da ativação, com adaptação bimodal, AASI e IC, observou-se localização lateral para direita e esquerda para sons instrumentais de média intensidade (guizo e reco-reco); reflexo cócleo-palpebral – RCP para som do agogô e reação à voz em intensidade habitual. Na avaliação da audiometria de reforço visual (VRA) os níveis mínimos de resposta encontrados foram: 500Hz – 45dB, 1000Hz – 60dB, 2000Hz – 65dB, 4000Hz – 70dB.

Além disso, a criança apresentou reação ao chamado do nome, atenção aos sons da voz e batida na porta, detecção dos sons /a/, /i/ e /u/, não sendo observada detecção para os sons /s/, /ʃ/ e /m/ durante prova com os sons do Ling.

Nesta ocasião, a partir da avaliação fonoaudiológica, a criança apresentava categoria auditiva 1 e categoria de linguagem 1.

### Caracterização dos controles

Participaram dessa pesquisa, para fins de comparação, três crianças saudáveis (de acordo com os registros médicos e relato dos responsáveis), pareadas de acordo com a idade auditiva da paciente implantada e sem indicadores de risco para perda auditiva, com presença de onda V a 35 dBnNA à triagem auditiva neonatal. Todas as crianças foram submetidas a avaliação auditiva, incluindo medidas de imitância acústica, potencial evocado auditivo de tronco encefálico automático a 35dBnNA (PEATEa) com estímulo CE-chirp e emissões otoacústicas evocadas por estímulos transientes (EOAT); apresentando resultados compatíveis com os padrões de normalidade para a faixa etária.

### Protocolo de coleta da fNIRS

Após a ativação do IC, na mesma data, foi feito o registro da fNIRS para avaliar a ativação cerebral frente à estimulação auditiva a partir do uso do dispositivo, conforme descrito adiante.

Para a pesquisa da atividade hemodinâmica cortical foi utilizada a técnica de espectroscopia de luz no infravermelho próximo - fNIRS. O equipamento utilizado para o estudo foi o *NIRScout Tandem 1616* (*NIRx MedicalTechnologies, Glen Head*). O protocolo desenvolvido utilizou comprimentos de onda de fontes de luz de 760 e 850 nm, com 30 fontes e 28 detectores, formando 84 canais que cobriam os lobos frontal, temporal, parietal e occipital bilateralmente, tomando como referência o sistema internacional 10-20 para posicionamento dos optodos. As distâncias entre as fontes e detectores foram de cerca de 3,5 cm. As Figuras 1 e 2 ilustram o protocolo de coleta desenvolvido para esse estudo.

**Figura 1:** Registro dos dados com a fNIRS

Legenda: 1a: equipamento usado para aquisição dos dados da fNIRS; 1b: imagem do registro da ativação cortical pessoal; 1c: geometria utilizada na disposição dos canais para o exame de espectroscopia

**Figura 2:** Representação visual da apresentação dos estímulos auditivos no tempo

Legenda: Em vermelho – áreas corticais ativadas durante estimulação

Na geometria utilizada, os 84 canais estão representados pelas linhas verdes.

Para a coleta das respostas corticais das crianças frente ao estímulo sonoro, o protocolo consistia em blocos com a estimulação da voz materna, em forma de conversa espontânea com prosódia direcionada para a criança. A estimulação sonora ocorreu durante seis vezes com duração média de 10 segundos cada, intercaladas com intervalos de silêncio de 10 segundos (Figura 2). Durante a tarefa, as crianças se encontravam no colo da mãe e a intensidade da emissão vocal ocorreu em torno de 60dBNPS, medidos com um decibelímetro manual.

### Protocolo de pré-processamento dos dados da fNIRS

Os dados do registro foram processados no *Brainstorm*, um aplicativo de código aberto para analisar gravações cerebrais como MEG, EEG e fNIRS^(12)^. O processamento do registro ocorreu com base no protocolo geral usado em experimentos anteriores de fNIRS^(13-15)^.

Para extrair a função de resposta hemodinâmica (HRF) para cada canal e rejeitar aqueles que não forneciam dados suficientes devido a ruído ou artefato de movimento^(13)^, foi necessário aplicar nos registros um procedimento de 10 etapas descritas a seguir: (1) Detectar e marcar manualmente artefatos de movimento caracterizados por variação de sinal com picos que se espalham para os canais e iniciar a correção de movimento semi automático com base na interpolação *spline* ^(16)^; (2) Detectar automaticamente canais ruins devido a valores negativos ou emparelhados ou devido a muitos segmentos planos usando uma proporção máxima de pontos de saturação e piso igual a 1,00; (3) Remover falhas (limiar de variação 2 vezes o desvio padrão); (4) Calcular variações de concentração de oxiemoglobina (HbO), desoxiemoglobina (HbR) e hemoglobina total (HbT) usando a Lei de Beer-Lambert modificada e configurar as opções de processo com referência de idade 1 ano, fator de volume parcial (PVF) igual a 6, método DPF^(17)^ e com base na média; (5) Remover automaticamente a tendência linear; (6) Usar filtro passa-banda de 0,02Hz e 0,8Hz para reduzir as flutuações hemodinâmicas e fisiológicas sistêmicas lentas e ruídos de instrumentos de alta frequência e oscilações rápidas; (7) Aplicar um filtro *notch* para eliminar a contaminação do sinal da rede elétrica em 30Hz; (8) Detectar e excluir automaticamente os canais usando códigos MATLAB, quando as mudanças de intensidade estiverem com relação sinal-ruído baixa (entre o desvio padrão e a média maior que 5 em uma janela de tempo de 5s para evitar gravação inadequada devido a cabelos grossos^(13)^; (9) Inspecionar visualmente os dados e remover canais com amplitude alta e variações de sinal durante a gravação dos blocos e; (10) Inspecionar visualmente todos os dados e remover das análises aqueles que ainda apresentam artefatos de movimentos.

A janela de tempo utilizada variou de -10.000 ms a +15.000 ms do início dos estímulos marcados pelos gatilhos. Para estabelecer um padrão, todas as amostras foram ajustadas para faixa de amostragem igual a 4,0Hz. Para normalização da linha de base, foi usada a transformação do Z-score selecionando a definição da linha de base de -5 a 0s. No final, foi feito o cálculo da média da função de resposta hemodinâmica por criança.

Para análise das concentrações de hemoglobina e desoxihemoglobina em resposta a ativação cortical foi utilizada a Lei de Beer-Lambert modificada. A lei consiste em uma equação que descreve a absorção da luz a um dado comprimento de onda em um meio, a lei foi modificada para levar em consideração a dispersão e a trajetória não linear da luz nos tecidos. Estes dois fatores não podem ser medidos usando a fNIRS, portanto apenas mudanças nas concentrações de HbO e HHb podem ser obtidas^(14)^.

Para análise estatística foi usado o teste Power F contra a linha de base, quando Y = sum_trials(X^2) e F = Y/tempo médio (Y (linha de base). A finalidade era encontrar alterações significativas, em relação à linha de base, na concentração de HbO e HbR em resposta a estímulos auditivos, apesar de valores negativos ou positivos.

Foi realizada a análise da resposta da criança implantada com três crianças ouvintes de mesma idade auditiva. Para comparar os grupos, foi utilizado o teste de Permutação, usando o teste de média absoluta (ou seja, T = (|média(A)|-|média(B)|) / sqrt(|var(A)|/nA + |var(B)|/nB)).

### Resultado fNIRS comparativo da criança implantada e das crianças ouvintes

As respostas foram calculadas considerando as medidas de Hbo e HHb resultante da atividade cortical em resposta às mudanças de oxigenação do sangue ao processar os estímulos oferecidos. As imagens a seguir mostram a resposta aos estímulos auditivos apresentados por meio da voz da mãe.

A Figura 3 mostra o *power test* da criança usuária do implante coclear (caso) com o registro da ativação frente a estimulação auditiva da voz da mãe. Os pontos em vermelho representam os pontos ativados.

**Figura 3:** *Power test* da ativação da criança implantada frente ao estímulo sonoro da voz da mãe

Legenda: Comparação entre criança implantada e crianças ouvintes – sem diferença significativa

É possível observar uma maior ativação na região temporal e parieto-occipital. Após analisar a resposta da criança implantada, foi utilizado o power test para comparar a atividade cerebral entre os grupos.

A Figura 4 mostra o *power test* da comparação entre a resposta da criança implantada com a resposta de três crianças de mesma idade auditiva frente ao estímulo da voz materna.

**Figura 4:** *Power test* do grupo das três crianças controle de mesma idade auditiva

A imagem em branco, fornecida pelo software, demonstra que não houve diferença em relação à linha de base (seja ela positiva ou negativa) neste grupo. Sugerindo que no grupo das crianças ouvintes, não houve diferença entre o período de estimulação e a linha de base deste mesmo grupo.

## DISCUSSÃO

No presente estudo, observou-se o desempenho auditivo após adaptação do implante coclear em uma criança com perda auditiva neurossensorial de grau profundo e também a dinâmica das áreas cerebrais ativadas com exame de neuroimagem. A resposta hemodinâmica cortical da criança com perda auditiva foi comparada com a resposta cortical de três crianças ouvintes da mesma idade auditiva.

O registro feito pela espectroscopia de luz no infravermelho próximo demonstrou maior ativação na região temporal em ambos os hemisférios e também na região occipital. Geralmente a região temporal é ativada para processar a prosódia e a fala, e a região occipital está relacionada ao processamento de estímulos visuais^(11)^. Estudos mostram que crianças precocemente implantadas ativam as mesmas áreas corticais dos indivíduos com audição normal em resposta aos sons, já as crianças implantadas tardiamente, ao ouvirem um som, ativam outras áreas corticais, não relacionadas a áreas auditivas^(18)^_._ Pode ser considerado um padrão difuso de resposta cortical em decorrência da limitação sensorial imposta pela perda auditiva.

A plasticidade neuronal é moldada pelas necessidades exigidas pelo meio^(2,18)^. Há diferentes períodos sensíveis para diferentes funções comportamentais, devido às estruturas e funções corticais^(18)^. A criança do estudo recebeu o implante coclear com 1 ano e 4 meses, dentro do período considerado sensível pela literatura^(19)^ e sem mudanças estruturais significativas no córtex, conforme observado pela comparação da fNIRS .

Neste período ocorre a plasticidade neuronal e a maturação das vias auditivas, proporcionando a aquisição das habilidades auditivas, de fala e de linguagem. No caso clínico do estudo, observamos que a criança iniciou o processo de restabelecimento da função auditiva em um período propício para favorecer o desenvolvimento cortical, ou seja, antes de completar 12 meses.

O desenvolvimento cerebral ocorre por meio das etapas de neurogênese, migração neuronal, desenvolvimento neuronal, eliminação e formação de caminhos centrais, gerando uma conexão cerebral funcional. A falta de estimulação sonora do córtex auditivo pode gerar o desacoplamento funcional do córtex primário e áreas auditivas secundárias^(2,20)^. A falta do *input* sensorial acarreta consequências desde o início da vida. A estimulação auditiva por meio do implante coclear no período sensível induz a plasticidade e a maturação, reduzindo as consequências negativas para o neurodesenvolvimento^(11,21)^.

As informações auditivas são processadas no cérebro por meio das funções de análise e categorização das características acústicas do som, essas funções são subjetivas e dependem do processo de aprendizagem. O estímulo sensorial favorece a reorganização dos campos receptivos do córtex cerebral adquirindo significados. O cérebro novo apresenta maior capacidade de reorganização e plasticidade neuronal comparado com cérebros já desenvolvidos^(19,22).^

Ao comparar a resposta da ativação da criança implantada com a ativação de crianças ouvintes o padrão de resposta se mostrou semelhante, o que nos permite inferir que já na ativação do implante coclear, quando ainda não foi atingida a meta alvo de audibilidade, a criança se mostrou com respostas hemodinâmicas corticais semelhantes aos seus pares, com audição normal, de mesma idade auditiva. Embora o fato de o caso aqui apresentado se tratar de uma perda auditiva pré-lingual, onde o fenômeno da *cross*-modalidade poderia ter acarretado mudanças estruturais no córtex da criança, a semelhança de respostas comparadas a seus pares ouvintes demonstra o quão importante é a intervenção no período sensível para o melhor desenvolvimento das funções auditivas e de linguagem da criança.

Um estudo populacional com crianças de surdez congênita mostrou que o benefício com o implante coclear se evidenciava quando as cirurgias ocorriam entre os primeiros 3,5 anos de vida, em que as vias auditivas centrais apresentam plasticidade máxima. Para confirmar os dados foram realizadas avaliações do potencial evocado auditivo cortical, onde foi possível observar a latência e a morfologia do componente P1 (considerado um biomarcador de maturação cortical). Houve diminuição da latência de P1 após a realização do implante coclear e os resultados se assemelham ao de crianças ouvintes. Além disso, os mesmos pesquisadores observaram nas crianças que receberam implantes antes dos 3-4 anos de idade melhor desempenho na percepção de fala e habilidades de linguagem quando comparadas a crianças implantadas após os 6-7 anos de idade^(23)^.

Importante ressaltar também que a criança estudada, além do uso do implante coclear, faz uso do AASI contralateral, promovendo o sistema de adaptação bimodal, isto é, o uso da estimulação elétrica via implante coclear associado com a estimulação acústica na outra orelha. Conforme relatado na literatura, esse modelo de adaptação fornece benefícios relacionados com a percepção de fala no ruído, localização sonora e outras situações de comunicação. Devido ao efeito da somação binaural, o cérebro percebe o som mais intenso exigindo menor concentração e maior facilidade de escuta pelo indivíduo^(2)^.

Embora testes subjetivos sejam amplamente utilizados na prática clínica e tem se mostrado confiáveis para mensurar as habilidades de audição e de linguagem de crianças surdas usuárias de AASI ou implante coclear, a compreensão do que acontece no nível cortical nesses indivíduos pode ampliar o conhecimento acerca do desenvolvimento auditivo com o uso dos dispositivos e a realização da fonoterapia.

Estudos mostram que crianças precocemente implantadas ativam as mesmas áreas corticais dos indivíduos com audição normal em resposta aos sons, já as crianças implantadas tardiamente ao ouvirem um som, ativam outras áreas de ativação cortical, não relacionadas a áreas auditivas^(18)^_._

Uma forma de entender como as atividades cerebrais acontecem e podem influenciar nos resultados do IC poderia auxiliar não só no prognóstico do sucesso desse procedimento, mas também monitorar as respostas pós-operatórias nesses pacientes, auxiliando na conduta em busca de melhores resultados^(5,6)^.

O uso da fNIRS possibilita o acesso à informação sobre a oxigenação cerebral, em especial das áreas de substância cinzenta com profundidade média de 3-4 cm^(11,24)^. A fNIRS permite demonstrar o aspecto funcional de ativação de uma determinada região cerebral diante de um estímulo específico. Por se tratar de tecnologia baseada exclusivamente em luz não há risco para o paciente e as limitações consistem apenas na fixação do eletrodo e no estabelecimento de bom sinal na captação^(6)^.

A neuroimagem, visualizada a partir da análise das respostas corticais captadas, auxilia no monitoramento do desempenho auditivo com o IC, pois a percepção auditiva ocorre além do córtex auditivo, e a imagem molecular, gerada pelas alterações na oxigenação cerebral (hemodinâmica cortical) fornece uma informação clínica adicional para avaliar se a estimulação elétrica do IC na cóclea está alcançando e estimulando regiões corticais auditivas específicas do cérebro semelhantes ao que ocorre em indivíduos com audição normal^(24,25)^.

Em um estudo com a aplicação da fNIRS em usuários de IC, observaram-se respostas corticais evocadas por fala em quatro grupos: adultos com audição normal, crianças com audição normal, crianças surdas que tiveram mais de quatro meses de audição por meio do implante coclear e crianças surdas após um mês do implante. Os estímulos de fala consistiam em gravações de histórias infantis em inglês. Os dados da fNIRS foram comparados com dados encontrados na ressonância magnética funcional e encontraram respostas semelhantes de ativação do giro temporal superior. Tais resultados demonstraram que a fNIRS era uma técnica de neuroimagem viável em usuários de IC e que respostas corticais hemodinâmicas confiáveis à fala poderiam ser registradas nesses pacientes^(26,27)^.

Em um estudo para avaliar a sensibilidade da fNIRS para detectar as diferenças na ativação cortical em resposta da qualidade da fala em indivíduos normais, foi utilizado um sistema fNIRS de 140 canais (NIRScout, NIRx Medical Technologies LLC, Glen Head, NY). O protocolo utilizado consistiu em quatro estímulos diferentes: fala normal, fala canalizada (simulando a distribuição por canais do implante coclear), fala embaralhada e ruído ambiental. Como resultados, os pesquisadores observaram que a fala normal provocou as respostas mais fortes, a fala distorcida produziu menos ativação específica da região e sons ambientais evocaram a menor resposta, sendo possível afirmar que a fNIRS pode detectar diferenças na resposta de inteligibilidade em indivíduos ouvintes^(10)^. Baseado nesse estudo foi feita uma pesquisa experimental abordando participantes com CI. Foi utilizado o instrumento NIRScout 1624 (NIRx Medical Technologies, LLC, Glen Head, NY) com 140 canais, para registrar a resposta auditiva cortical de 32 adultos surdos pós-linguais ouvindo através de um IC e 35 adultos com audição normal. Os mesmos estímulos foram adotados como protocolo: fala normal, fala canalizada, fala embaralhada e ruído ambiental. Nesse experimento foi possível observar que o padrão de ativação cortical em adultos implantados com boa percepção de fala foi semelhante ao dos controles. Os resultados deste estudo demonstraram que os padrões de ativação no córtex auditivo dos receptores de IC estão correlacionados com a qualidade da percepção de fala. A avaliação foi repetida com o implante desligado, e foram observadas ativações corticais reduzidas em todos os receptores de IC^(10)^.

A técnica e o registro de fNIRS, também podem ser realizados em conjunto com a eletroencefalografia e um estudo com crianças implantadas identificou que a modalidade cruzada nestas crianças não é tão especializada e uniforme quanto esperado^(28)^.

Em crianças ouvintes, observa-se que, mesmo em bebês, há ativação cortical bilateral frente à estimulação sonora e as respostas à voz materna estão presentes ainda de forma difusa e sem diferença em relação a outros estímulos^(29)^.

Todos estes estudos mostram como é vasta a aplicação do estudo das respostas corticais para aprofundar a compreensão do desenvolvimento auditivo.

Novas pesquisas relacionadas à avaliação do NIRS concomitante ao uso do implante coclear devem ser desenvolvidas.

Considerando as diversas técnicas de coleta, processamento e análise de resultados encontradas nas pesquisas realizadas, torna-se necessário o desenvolvimento de protocolos para validar os registros e interpretações da NIRS com o IC, favorecendo a compreensão dos aconselhamentos e reabilitação aos pacientes antes e após a implantação^(10)^. A fNIRS pode no futuro se tornar um teste pré-implante e pós-implante, para fornecer informações clínicas importantes relacionadas ao prognóstico e intervenção dos pacientes, podendo fornecer suporte para as terapias individuais e potencializar os seus resultados^(24)^.

## CONSIDERAÇÕES FINAIS

Este relato de caso comparativo descreveu as respostas auditivas após ativação do implante coclear em uso concomitante do AASI. Observou-se respostas corticais com a finalidade de entender as mudanças ocorridas no cérebro da criança com perda auditiva implantada em comparação ao cérebro de crianças ouvintes.

Os resultados encontrados sugerem mais uma vez que a realização do implante coclear em tempo oportuno, onde o córtex ainda não está totalmente especializado para processar determinadas tarefas, é importante para o máximo aproveitamento da plasticidade neuronal, favorecendo o desenvolvimento das habilidades auditivas e de linguagem.

Além disso, a fNIRS se mostrou novamente uma ferramenta interessante para ser utilizada em usuários de implante coclear.

A aplicação destes achados na população pediátrica usuária de implante coclear é muito promissora para detectar e monitorar o prognóstico e reabilitação desses indivíduos. O estudo está sendo ampliado para um maior número de crianças e variadas condições de pareamento para fins comparativos. Sua metodologia pode ser reproduzida em outros estudos com população pediátrica na observação do desenvolvimento auditivo decorrente da reabilitação auditiva.
